# Evaluation of research co-design in health: a systematic overview of reviews and development of a framework

**DOI:** 10.1186/s13012-024-01394-4

**Published:** 2024-09-11

**Authors:** Sanne Peters, Lisa Guccione, Jill Francis, Stephanie Best, Emma Tavender, Janet Curran, Katie Davies, Stephanie Rowe, Victoria J. Palmer, Marlena Klaic

**Affiliations:** 1 School of Health Sciences, The University of Melbourne, Melbourne, Australia; 2 Department of Health Services Research, Peter MacCallum Cancer Centre, Melbourne, Australia; 3 Sir Peter MacCallum Department of Oncology, Faculty of Medicine, Dentistry and Health Sciences, The University of Melbourne, Melbourne, Australia; 4 Centre for Implementation Research, Ottawa Hospital Research Institute, Ottawa, Canada; 5 Victorian Comprehensive Cancer Centre, Melbourne, VIC Australia; 6 Emergency Research, Murdoch Children’s Research Institute, Melbourne, Australia; 7 Department of Critical Care, The University of Melbourne , Melbourne, Australia; 8 School of Nursing, Faculty of Health, Ottawa, Canada; 9 Emergency Medicine, Faculty of Medicine, Ottawa, Canada; 10 Neurological Rehabilitation Group Mount Waverley, Mount Waverley, Australia; 11 The ALIVE National Centre for Mental Health Research Translation, The University of Melbourne, Melbourne, Australia

**Keywords:** Research co-design, Evaluation, Stakeholder involvement, End-user engagement, Consumer participation, Outcome measures

## Abstract

**Background:**

Co-design with consumers and healthcare professionals is widely used in applied health research. While this approach appears to be ethically the right thing to do, a rigorous evaluation of its process and impact is frequently missing. Evaluation of research co-design is important to identify areas of improvement in the methods and processes, as well as to determine whether research co-design leads to better outcomes. We aimed to build on current literature to develop a framework to assist researchers with the evaluation of co-design processes and impacts.

**Methods:**

A multifaceted, iterative approach, including three steps, was undertaken to develop a Co-design Evaluation Framework: 1) A systematic overview of reviews; 2) Stakeholder panel meetings to discuss and debate findings from the overview of reviews and 3) Consensus meeting with stakeholder panel. The systematic overview of reviews included relevant papers published between 2000 and 2022. OVID (Medline, Embase, PsycINFO), EBSCOhost (Cinahl) and the Cochrane Database of Systematic reviews were searched for papers that reported co-design evaluation or outcomes in health research. Extracted data was inductively analysed and evaluation themes were identified. Review findings were presented to a stakeholder panel, including consumers, healthcare professionals and researchers, to interpret and critique. A consensus meeting, including a nominal group technique, was applied to agree upon the Co-design Evaluation Framework.

**Results:**

A total of 51 reviews were included in the systematic overview of reviews. Fifteen evaluation themes were identified and grouped into the following seven clusters: People (within co-design group), group processes, research processes, co-design context, people (outside co-design group), system and sustainment. If evaluation methods were mentioned, they mainly included qualitative data, informal consumer feedback and researchers’ reflections. The Co-Design Evaluation Framework used a tree metaphor to represent the processes and people in the co-design group (below-ground), underpinning system- and people-level outcomes beyond the co-design group (above-ground). To evaluate research co-design, researchers may wish to consider any or all components in the tree.

**Conclusions:**

The Co-Design Evaluation Framework has been collaboratively developed with various stakeholders to be used prospectively (planning for evaluation), concurrently (making adjustments during the co-design process) and retrospectively (reviewing past co-design efforts to inform future activities).

**Supplementary Information:**

The online version contains supplementary material available at 10.1186/s13012-024-01394-4.

Contributions to the literature

- While stakeholder engagement in research seems ethically the right thing to do, a rigorous evaluation of its process and outcomes is frequently missing.
- Fifteen evaluation themes were identified in the literature, of which *research process*, *cognitive* and *emotional factors* were the most frequently reported.
- The Co-design Evaluation Framework can assist researchers with research co-design evaluation and provide guidance regarding what and when to evaluate.
- The framework can be used prospectively, concurrently, and retrospectively to make improvements to existing and future research co-design projects.

## Introduction

Lots of money is wasted in health research that does not lead to meaningful benefits for end-users, such as healthcare professionals and consumers [1–3]. One contributor to this waste is that research often focusses on questions and outcomes that are of limited importance to end-users [4, 5]. Engaging relevant people in research co-design has increased in order to respond to this issue. There is a lack of consensus in the literature on the definition and processes involved in undertaking a co-design approach. For the purposes of this review, we define research co-design as *meaningful* end-user *engagement* that occurs across *any stage of the research process*, from the research planning phase to dissemination of research findings [6]. Meaningful end-user engagement refers to an explicit and measurable responsibility, such as contributing to writing a study proposal [6]. The variety of research co-design methods can be seen as a continuum ranging from limited involvement, such as consulting with end-users, to the much higher effort research approaches in which end-users and researchers aim for equal decision-making power and responsibility across the entire research process [6]. Irrespective of the intensity of involvement, it is generally recommended that a co-design approach should be based on several important principles such as equity, inclusion and shared ownership [7].

Over time, increasing attention has been given to research co-design [6, 8]. Funding bodies encourage its use and it is recommended in the updated UK MRC framework on developing and evaluating complex interventions [9]. End-user engagement has an Equator reporting checklist [10] and related work has been reported by key organisations, such as the James Lind Alliance in the UK (www.jla.nihr.ac.uk), Patient Centered Outcomes Research Institute in the US (www.pcori.org) and Canadian Institutes of Health Research (https://cihrirsc.gc.ca/e/41592.html). In addition, peer reviewed publications involving co-design have risen from 173 per year in 2000 to 2617 in 2022 (PubMed), suggesting a growing importance in research activities.

Engaging end-users in the health research process is arguably the right thing to do, but the processes and outcomes of co-design have rarely been evaluated in a rigorous way [6]. Existing anecdotal evidence suggests that research co-design can benefit researchers, end-users and lead to more robust research processes [11–19]. Both researchers and end-users have reported positive experiences of engaging in the co-design process. Potential benefits include a better understanding of community needs, more applicable research questions, designs and materials and improved trust between the researchers and end-users. Several reviews on conducting research co-design have concluded that co-design can be feasible, though predominantly used in the early phases of research, for example formulating research questions and developing a study protocol [6, 11–19]. However, these reviews highlighted that engagement of end-users in the research process required extra time and funding and had the risk of becoming tokenistic [6, 11–19].

The use of resources in co-design studies might need to be justified to the funder as well as its impacts. A rigorous evaluation of research co-design processes and outcomes is needed to identify areas of potential improvement and to determine the impact of research co-design. Several overviews of reviews on research co-design have been published but with no or limited focus on evaluation [20–23]. Moreover, current literature provides little guidance around how and what to evaluate, and which outcomes are key.

This study thus had two aims:

1. To conduct a systematic overview of reviews to identify evaluation methods and process and outcome variables reported in the published health research co-design literature.
2. To develop a framework to assist researchers with the evaluation of co-design processes and impacts.

## Methods

This project used a multifaceted, iterative approach to develop a Co-design Evaluation Framework. It consisted of the following steps: 1) A systematic overview of reviews; 2) Stakeholder panel meetings to discuss and debate findings from the overview of reviews and 3) Consensus meeting with stakeholder panel. The reporting checklist for overviews of reviews was applied in Additional file 1 [24].

### Step 1: A systematic overview of reviews

We conducted a systematic overview of reviews [25], reviewing literature reviews rather than primary studies, to investigate the following question: What is known in the published literature about the evaluation of research co-design in health research? The protocol of our systematic overview of reviews was published in the PROSPERO database (CRD42022355338).

Sub questions:

- What has been co-designed and what were the objectives of the co-design process?
- Who was involved and what was the level of involvement?
- What methods were used to evaluate the co-design processes and outcomes?
- What was evaluated (outcome and process measures) and at what timepoint (for example concurrently, or after, the co-design process)?
- Was a co-design evaluation framework used to guide evaluation?

#### Search strategy

We searched OVID (Medline, Embase, PsycINFO), EBSCOhost (Cinahl) and the Cochrane Database of Systematic reviews on the 11th of October 2022 for literature reviews that reported co-design evaluation or outcomes in health research. The search strategy was based on previous reviews on co-design [6, 14, 26] and refined with the assistance of a research librarian and the research team (search terms in Additional file 2). Papers published from January 2000 to September 2022 were identified and retrieved by one author (SP).

#### Study selection

Database records were imported into EndNote X9 (The EndNote Team, Philadelphia, 2013) and duplicates removed. We managed the study selection process in the software program Covidence (Veritas Health Innovation, Melbourne, Australia). Two independent reviewers (SP, MK or LG) screened the titles and abstracts of all studies against the eligibility criteria (Table 1). Discrepancies were resolved through discussion or with a third reviewer (either SP, MK or LG, depending on which 2 reviewers disagreed). If there was insufficient information in the abstract to decide about eligibility, the paper was retained to the full-text screening phase. Full-text versions of studies not excluded at the title and abstract screening phase were retrieved and independently screened by two reviewers (SP, MK or LG) against eligibility criteria. Disagreements were resolved through discussion, or with a third reviewer, and recorded in Covidence.

**Table 1:** Inclusion and exclusion criteria for the overview of reviews

Inclusion criteria	Exclusion criteria
• Any kind of literature review, or paper that contains a literature review as part of a multi-method paper, that reported on evaluation or outcomes of research co-design in health (or any synonym of research co-design) • Research co-design with end-users including any individual or group who may potentially receive the healthcare intervention (e.g., consumers, family/carers), and/or deliver the intervention (e.g., healthcare professionals), and others who are impacted by the healthcare intervention (e.g., health managers, administration staff, funding bodies) • Papers on guideline implementation were included if they focused on research (rather than solely implementation practice) and consumers and/or healthcare professionals were engaged in co-design	• Papers not available in English • Reviews published in journals that are not peer-reviewed (including reviews published in books) • Reviews of solely non-published reports • Grey literature • Papers with no formal literature review (if there was insufficient data reported on methods and findings of the review) • Papers that solely reported on stakeholders as participants in research • Papers about students who are being trained to become a healthcare professional • Co-design with children (up to 18 years old) • Papers about health policy, health service development and public health initiatives, such as public health campaigns • Overviews of reviews

Data extraction of included papers was conducted by one of three reviewers (SP, MK or LG). A second reviewer checked a random sample of 20% of all extracted data (LG or SP). Disagreements were resolved through regular discussion. Data were extracted using an excel spreadsheet developed by the research team and included review characteristics (such as references, type of review, number of included studies, review aim), details about the co-design process (such as who was involved in the co-design, which topics the co-design focused on, what research phase(s) the co-design covered, in which research phase the co-design took place and what the end-users’ level of involvement was) and details about the co-design evaluation (what outcomes were reported, methods of data collection, who the participants of the evaluation were, the timepoint of evaluation, whether an evaluation framework was used or developed and conclusions about co-design evaluation).

Types of end-users’ involvement were categorised into four groups based on the categories proposed by Hughes et al. (2018): 1. Targeted consultation; 2. Embedded consultation; 3. Collaboration and co-production and 4. User-led research, see Table 2.

**Table 2:** Types of end-users’ involvement (adapted from [27])

Type	Explanation
Targeted consultation	People are contacted and consulted on specific aspects of the study, for example tasks such as a research proposal, wording of information sheets or surveys. Those involved may not be otherwise involved in the design of the study and may not receive much information regarding subsequent progress, outputs or impact
Embedded consultation	People with relevant lived experience are consulted regularly throughout the research cycle from initial ideas and proposals to dissemination of findings. People may be individual representatives on steering or advisory groups or be representing a user-led organisation. The research team retains ownership and control over the research study with regular input from the public
Collaboration and co-production	People with relevant lived experience are active members of the research team, contributing to key decisions regarding the research process as well as the findings. Relationships are reciprocal and collaborative, with shared control across researchers, patients and the public, based on specific areas of expertise
User-led research	People with lived experience are supported to lead the research, and take a systematic approach to redirecting the team through each stage from selecting the topic, writing proposals, designing the intervention, collecting and analysing data, and disseminating findings

**Fig. 1:**
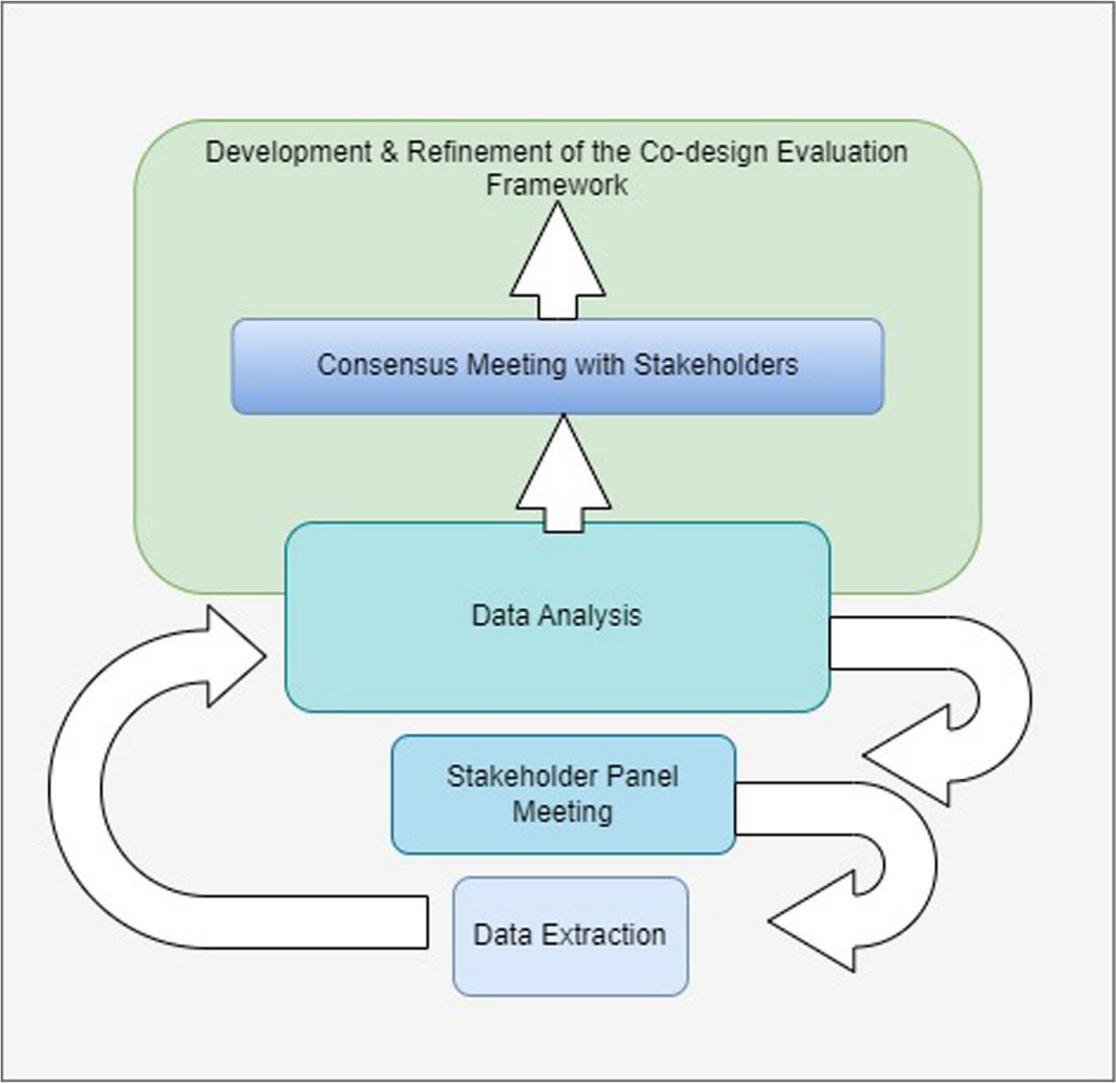
Iterative phases in the process of the Co-design evaluation framework development

Data extraction and analysis took place in three iterative phases (Fig. 1), with each phase containing one third of the included studies. Each phase of data extraction and analysis was followed by stakeholder panel meetings (see step 2 below). This stepwise approach enabled a form of triangulation wherein themes that emerged through each phase were discussed with the stakeholder panel and incorporated both retrospectively (re-coding data in the prior phase) and prospectively (coding new data in the next phase).

All reported outcomes of research co-design in the first phase (one third of all data) were inductively coded into themes, according to the principles of thematic analysis [28]. Two researchers (SP and MK) double coded 10% of all data and reached consensus through discussion. Given that consensus was high, one researcher (SP) continued the coding while having frequent discussions and reviews within the research team. In phase 2 (also one third of all data), deductive coding was based on the themes identified in the first round. Data of the first phase were re-coded, if new codes emerged during the stakeholder panel meeting. The same process took place for the third phase.

### Step 2: Stakeholder panel meetings to discuss and debate findings from the overview of reviews

Results from step 1 were presented to the stakeholder panel to interpret and critique the review findings. The panel consisted of ten people, including a mix of consumers, healthcare professionals and researchers. Stakeholders were selected for their experience or expertise in research co-design. The number of meetings was not pre-determined, rather, it was informed by the outcomes from step 1. The number of stakeholders in each meeting ranged from six to ten.

A core group from the broader stakeholder panel (SP, MK, LG, JF) with a breadth of research experience and methodological expertise discussed the themes arising from both steps 1 and 2 and considered various ways of presenting them. Multiple design options were considered and preliminary frameworks were developed. Following discussion with the stakeholder panel, it was agreed that the evaluation themes could be grouped into several clusters to make the framework more comprehensible. The grouping of evaluation themes into clusters was informed by reported proposed associations between evaluation themes in the literature as well as the stakeholder panel’s co-design experience and expertise. Evaluation themes as well as clusters were agreed upon during the stakeholder panel meetings.

### Step 3: Consensus meeting with stakeholder panel

The consensus meeting included the same stakeholder panel as in step 2. The meeting was informed by a modified Nominal Group Technique (NGT). The NGT is a structured process for obtaining information and reaching consensus with a target group who have some association or experience with the topic [29]. Various adaptations of the NGT have been used and additional pre-meeting information has been suggested to enable more time for participants to consider their contribution to the topic [30]. The modified NGT utilised in this study contained the following: (i) identification of group members to include experts with depth and diverse experiences. They were purposively identified at the start of this study for their expertise or experience in research co-design and included: a patient consumer, a clinician, three clinician researchers and six researchers with backgrounds in behavioural sciences, psychology, education, applied ethics and participatory design. All authors on this paper were invited by e-mail to attend an online meeting; (ii) provision of information prior to the group meeting included findings of the overview of reviews, a draft framework and objectives of the meeting. Five authors with extensive research co-design experience were asked to prepare a case example of one of their co-design projects for sharing at the group meeting. The intention of this exercise was to discuss the fit between a real-world example and the proposed framework; (iii) hybrid meeting facilitated by two researchers (SP & JF) who have experience in facilitating consensus meetings. Following presentation of the meeting materials, including the preliminary framework, group members were invited to silently consider the preliminary framework and generate ideas and critiques; iv) participants sharing their ideas and critiques; v) clarification process where group members shared their co-design example project and discussed the fit with components of the initial framework, and vi) silent voting and/or agreement on the framework via a personal email to one of the researchers (SP).

## Results

### Step 1: Systematic overview of reviews

**Fig. 2:**
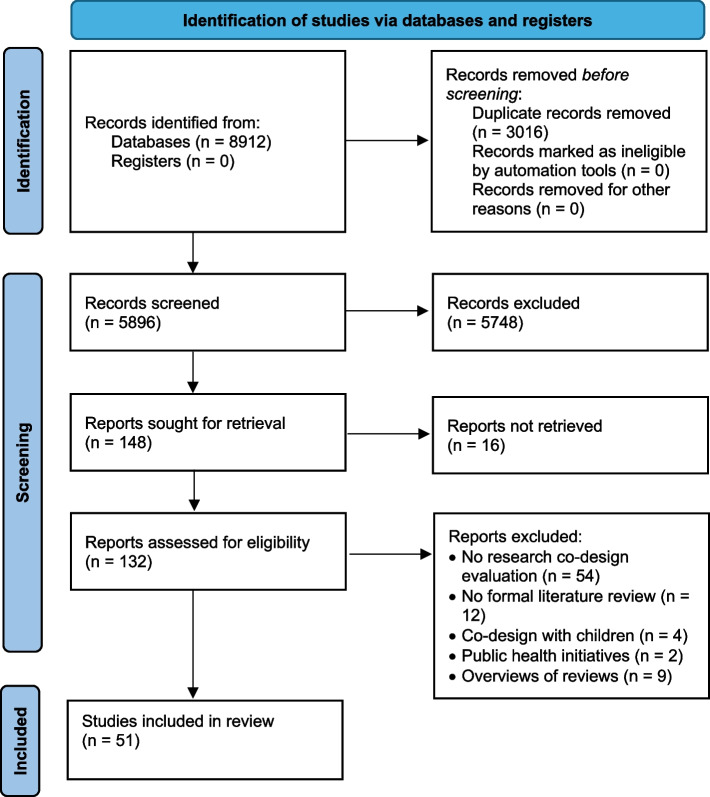
PRISMA flow chart (based on [31]) of overview of reviews

The database searches identified a total of 8912 papers. After removing 3016 duplicates and screening 5896 titles and abstracts, 148 full texts were sought for retrieval. Sixteen were not retrieved as they were not available in English (*n* = 2) or full-text was not available (*n* = 14). Of the remaining 132 papers assessed for eligibility, 81 were excluded. The final number of papers included in this overview of reviews was 51 (See Fig. 2).

#### Characteristics of the included studies

Of the 51 included reviews [11, 12, 14, 32–79], 17 were systematic reviews, 12 were scoping reviews, 14 did not report the type or method of review, three were narrative reviews, two were qualitative evidence synthesis, another two were a structured literature search and one was a realist review. The number of studies included in the reviews ranged from 7 to 260. Nineteen reviews focused on co-design with specific populations, for example older people, people with intellectual disabilities, people living with dementia and 32 reviews included co-design with a range of end-users. The co-design focused in most cases on a mix of topics (*n* = 31). Some reviews were specifically about one clinical topic, for example critical care or dementia. In ten cases, the clinical topics were not reported. Co-design took place during multiple research phases. Thirty-six reviews covered co-design in agenda/priority setting, 36 in study design, 30 in data collection, 25 in data analysis and 27 in dissemination. With regards to the research translation continuum, most of the co-design was reported in practice and community-based research (*n* = 32), three reviews were conducted in basic research and 11 in human research. The types of end-users’ involvement in co-design ranged from targeted consultation (*n* = 14) to embedded consultation (*n* = 20), collaboration and co-production (*n* = 14) to end-user- led research (*n* = 6), including papers covering multiple types of involvement. Seventeen papers did not report the types of involvement. The reported co-design included a variety of time commitments, from a minimum of a one-off 60-min meeting to multiple meetings over multiple years. Twenty-seven reviews did not report details about the end-users’ types of involvement.

### Step 2: Stakeholder panel meetings to discuss and debate findings from the overview of reviews

#### Identified evaluation themes

Fifteen evaluation themes were identified and were arranged into two higher level groups: 1. within the co-design team and 2. broader than co-design team (Table 3). The themes related to the first group (within the co-design team) included: Structure and composition of the co-design group, contextual enablers/barriers, interrelationships between group members, decision making process, emotional factors, cognitive factors, value proposition, level/ quality of engagement, research process, health outcomes for co-design group and sustainment of the co-design team or activities. The themes within the second group (broader than co-design team) included: Healthcare professional-level outcomes, healthcare system level outcomes, organisational level outcomes and patient and community outcomes.

**Table 3:** Evaluation themes identified in the overview of reviews

Theme	Definition	Examples
WITHIN CO-DESIGN TEAM		
Structure and composition of the co-design group	Outcomes of the recruitment and establishment process of a co-design team and group operations	· Members’ characteristics, experiences and areas of expertise · Objectives of the co-design group and who was involved in the development of these objectives
Contextual enablers/barriers	Outcomes that refer to situational aspects that might enable or hinder consumer engagement	· Funding (reimbursement for time and costs related to facilitating co-design) · Clear and jargon-free communication that is appropriate for the consumers’ circumstances · Adaptations to meet consumers’ needs, e.g. accessible buildings · Flexible meeting times
Interrelationships between group members	Outcomes related to relationship building processes within the co-design stakeholder group, from the perspective of the consumers and/or the researchers	· Belonging to a group · Mutual support · Trust · Solidarity · Group cohesion · Conflict · Communication
Decision making process	Outcomes related to how decisions are being made within the co-design group	· Power · Psychological safety · Feeling listened to · Leadership · Consensus
Emotional factors	Personal and emotional outcomes about being involved in the co-design process. These outcomes relate to consumers and/or researchers	· Confidence · Enjoyment · Being valued · Feeling empowered · Satisfaction
Cognitive factors	Outcomes related to strategies to support the person to acquire knowledge and understanding	· Knowledge · Skills · Attitudes about doing research · Attitudes about the study topic
Value-proposition	Outcomes referring to an analysis of pros and cons of consumer engagement	· Whether the additional effort and resources are seen as worthwhile by consumers · Whether the additional effort and resources are seen as worthwhile by researchers
Level/ quality of engagement	Outcomes related to consumers’ and researchers’ investment of effort and meaningful contributions in co-design	· Attendance rates at co-design activities · Active engagement versus tokenistic consumer involvement · The whole spectrum from a one-off meeting to substantial contributions during meaningful interactions between researchers and consumers
Research process	Outcomes related to: · Research quality · Relevance of the research to local context and the target population · Efficiency of the research process	· New ideas/perspectives, improved intervention fidelity, more feasible study design and acceptable data collection plans for consumers and/or researchers · Research questions based on local needs, reading level of study materials appropriate for setting and communication to participants/community adjusted to context · Consumers helped with participant recruitment or dissemination of study findings or less efficient (time consuming)
Health outcomes for co-design group	Any health outcomes related to the consumers who were part of the co-design team	· Improved health · Improved well-being
Sustainment of the co-designed interventions or co-design team beyond the current project	Outcomes of sustainment of the co-design group or co-design activities after the conclusion of the research project	· The team continued to work together on a different project · A team member continued to stay involved in a role related to the co-design process
BROADER THAN CO-DESIGN TEAM		
Healthcare professional-level outcomes	Any outcomes related to healthcare professionals who were not part of the research co-design (where this is relevant)	· Knowledge about study topic · Applying research findings in practice · More/less evidence-based care
Healthcare system-level outcomes	Outcomes related to changes in healthcare systems • Nation • State/ province	· Change in policy · Change in model of care · More/less evidence-based care · Funding for workforce
Organisational-level outcomes	Any outcomes related to change at the organisational level. An organisation could be a hospital or an aged care facility, for example	· Change in policy · Organisational leadership support · More/less evidence-based care
Patient and community outcomes	Any outcomes related to the research consumers who were participants in the research (but not part of the co-design team) and consumers in broader community	· Community trust in research · Improved health outcomes

The *research process* was the most frequently reported evaluation theme in the reviews (*n* = 44, 86% of reviews), followed by *cognitive factors* (*n* = 35, 69%) and *emotional factors* (*n* = 34, 67%) (Table 4). Due to variability in reporting practices, it was not possible to specify the number of primary studies that reported specific evaluation themes. Evaluation methods for the themes were not reported in the majority of reviews (*n* = 43, 84%). If evaluation methods were mentioned, they were mainly based on qualitative data, including interviews, focus groups, field notes, document reviews and observations (see overview with references in Additional file 3). Survey data was mentioned in three reviews. Many reviews reported informal evaluation based on participant experiences (e.g. informal feedback), reflection meetings, narrative reflections and authors’ hypotheses (Additional file 3). The timing of the evaluation was only mentioned in two papers: 1. Before and after the co-design activities and 2. Post co-design activities. One paper suggested that continuous evaluation might be helpful to improve the co-design process (Additional file 3).

**Table 4:** Reported evaluation themes and measurement methods

	**Number of reviews (%)**
**Reported outcomes**	
• Research process	44 (86%)
• Cognitive factors	35 (69%)
• Emotional factors	34 (67%)
• Interrelationships between group members	30 (59%)
• Patient and community outcomes	25 (49%)
• Structure and composition of the co-design group	22 (43%)
• Decision making process	19 (37%)
• Contextual enablers/barriers	16 (31%)
• Level/quality of engagement	14 (27%)
• Organisational- level outcomes	7 (14%)
• Sustainment of the co-design team or activities	7 (14%)
• Healthcare professional-level outcomes	6 (12%)
• Healthcare system-level outcomes	6 (12%)
• Value-proposition	2 (4%)
• Health outcomes for co-design group	1 (2%)
**Measurement methods for reported outcomes**	
• Not reported	43 (84%)
• Participant experiences (e.g. informal feedback)	5 (10%)
• Interviews	4 (8%)
• Surveys	3 (6%)
• Authors’ hypotheses	3 (6%)
• Focus groups	2 (4%)
• Reflection meetings	1 (2%)
• Narrative reflections	1 (2%)
• Field notes	1 (2%)
• Document reviews	1 (2%)
• Observations	1 (2%)

The systematic overview of reviews found that some authors reported proposed positive associations between evaluation themes (Table 5). The most frequently reported proposed association was between level/quality of engagement and emotional factors (*n* = 5, 10%). However, these proposed associations did not seem to have any empirical evidence and evaluation methods were not reported.

**Table 5:** Reported proposed associations between evaluation themes

	**Number of reviews (%)**
**Reported proposed associations between evaluation themes**	
• Level/quality of engagement and emotional factors	• 5 (10%)
• Level/quality of engagement and interrelationships between group members	• 3 (6%)
• Cognitive factors and emotional factors	• 2 (4%)
• Level/quality of engagement and research process	• 2 (4%)
• Level/quality of engagement and cognitive factors	• 2 (4%)
• Level/quality of engagement and healthcare system-level outcomes	• 1 (2%)
• Level/quality of engagement and contextual enablers/barriers	• 1 (2%)
• Level/quality of engagement and value-proposition	• 1 (2%)
• Level/quality of engagement and decision making process	• 1 (2%)
• Decision making process and emotional factors	• 1 (2%)
• Emotional factors and research process	• 1 (2%)
• Interrelationships between group members and research process	• 1 (2%)
• Decision making process and research process	• 1 (2%)
• Decision making process and patient and community outcomes	• 1 (2%)
**Measurement methods for reported proposed associations**	
• Not reported	100%

All evaluation themes were grouped into the following clusters (Table 6): People (within co-design group), group processes, research processes, co-design context, people (outside co-design group), system and sustainment.

**Table 6:** Clusters of evaluation themes

**Clusters**	**Evaluation themes**
People (within co-design group)	• Emotional factors • Cognitive factors • Value-proposition • Health outcomes for co-design group
Group processes	• Structure and composition of the group • Interrelationships between group members • Decision making processes • Level/quality of engagement • Sustainment of co-design team
Research processes	• Research quality • Relevance of the research to local context and the target population • Efficiency of the research process
Co-design context	• Contextual enablers/barriers
People (outside co-design group)	• Healthcare professional-level outcomes • Patient and community outcomes
System	• Healthcare system- level outcomes o Nation o State/ province • Organisational- level outcomes
Sustainment	• Sustainment of co-designed interventions (incl. adaptations as required) • Sustainment of co-design team beyond the current project • Sustainment of people-level outcomes • Sustainment of system-level outcomes

Only one paper reported the evaluation in connection to the research phases (Agenda/priority setting, study design, data collection, data analysis and dissemination). This paper reported the following outcomes for the following research phases [58]:

- Agenda/priority setting: Research process; Level/quality of engagement; Cognitive factors; Attributes of the co-design group; Interrelationships between group members; Sustainment of the co-design team or activities; Patient and community outcomes.
- Study design: Attributes of the co-design group; Interrelationships between group members; Level/quality of engagement; Cognitive factors; Emotional factors; Research process.

The various research phases in which consumers could be involved, as well as the clusters of evaluation themes, informed the design of the co-design evaluation framework.

### Step 3: Consensus meeting with stakeholder panel

Two main options were voted on and discussed within the stakeholder panel. The two main options can be found in Additional file 4. Draft 2 was the prefered option as it was perceived as more dynamic than draft 1, representing a clearer interplay between the two contexts. The stakeholder panel suggested a few edits to the draft, such as the inclusion of bi-directional arrows in the tree trunk and a vertical arrow from underground to above ground with the label ‘impact’.

**Fig. 3:**
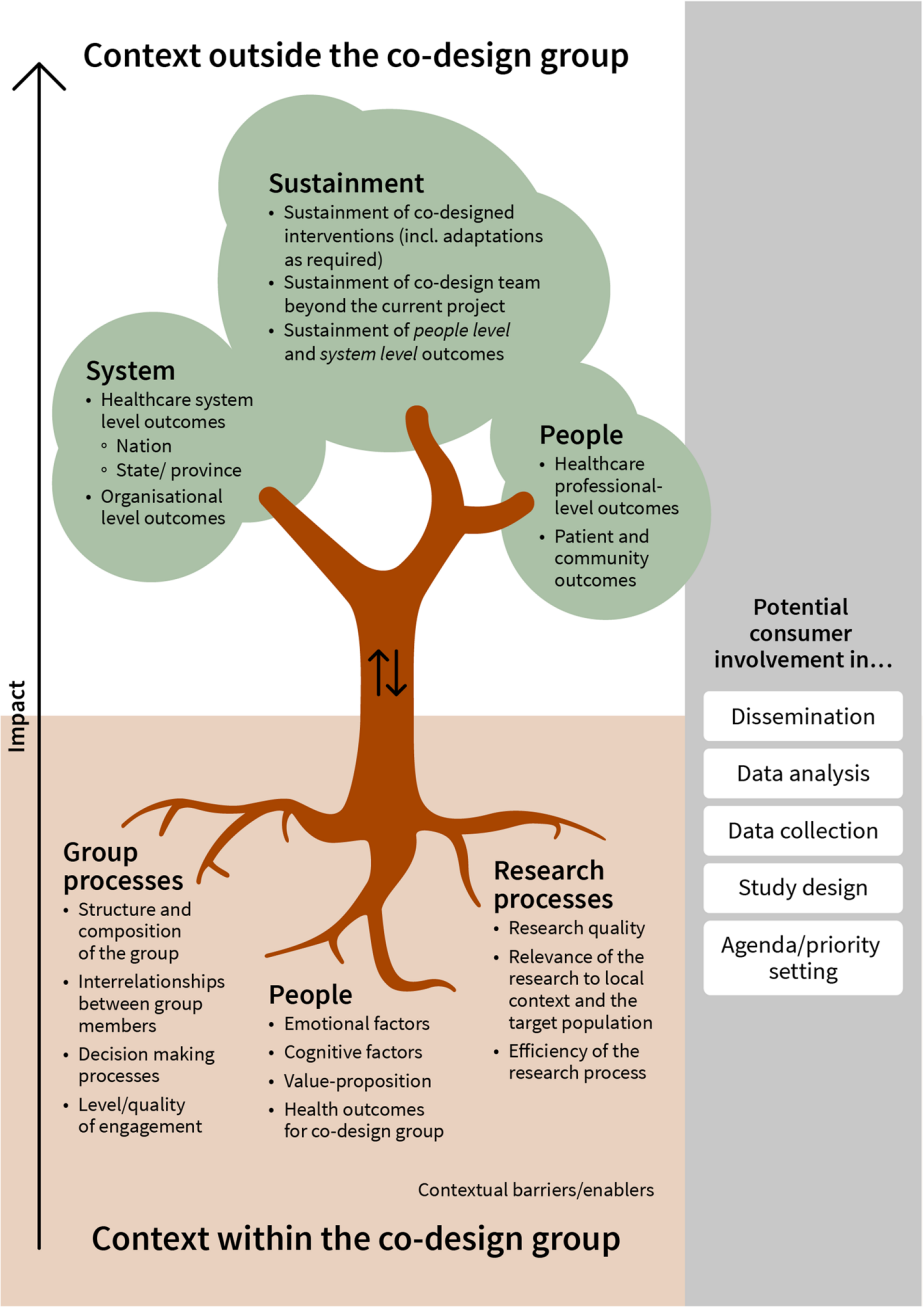
Research Co-design evaluation framework

The final version of the Co-design Evaluation framework is presented in Fig. 3.

Figure 3 presents co-design evaluation as the below-ground and above-ground structures of a tree. The tree metaphor presents the processes and people in the co-design group (below-ground) as the basis for system- and people-level outcomes beyond the co-design group (above-ground). To evaluate research co-design, researchers may wish to consider any or all components in this Figure. These evaluation components relate to the methods, processes, and outcomes of consumer involvement in research.

The context within the co-design group (the roots of the tree) consists of the people, group processes and research processes, with various evaluation themes (dot points) related to them, as well as contextual barriers and enablers that relate to situational aspects that might enable or hinder consumer engagement. The context outside the co-design group, i.e., the wider community (the branches and leaves of the tree), comprises people who were not involved in the research co-design process, the system-level and sustainment-related outcomes. These above ground groups are potential beneficiaries or targets of the co-design activities.

The arrows in the middle of the trunk represent the potential mutual influence of the two contexts, suggesting that an iterative approach to evaluation might be beneficial. For example, when deciding the composition of the co-design group, it may be important to have an appropriate representation of the people most impacted by the problem issue or topic at hand. Or, if a co-designed healthcare intervention does not achieve the desired outcomes in the wider context, the co-design group might consider potential ways to improve the intervention or how it was delivered. Evaluation of a research co-design process might start with the foundations (the roots of the tree) and progress to above ground (the tree grows and might develop fruit). Yet, depending on the aim of the evaluation, a focus on one of the two contexts, either below or above ground, might be appropriate.

Which, and how many, components are appropriate to evaluate depends on the nature of the co-design approach and the key questions of the evaluation. For example, if a co-design approach is used in the very early stages of a research program, perhaps to identify priorities or to articulate a research question, then 'below' the ground components are key. While a randomised study comparing the effects of a co-designed intervention versus a researcher-designed intervention might only consider 'above' the ground components.

The white boxes on the right-hand side of Fig. 3 indicate the research phases, from agenda/priority setting to dissemination, in which consumers can and should be involved. This co-design evaluation framework may be applied at any phase of the research process or applied iteratively with a view to improving future co-design activities.

## Discussion

This systematic overview of reviews aimed to build on current literature and develop a framework to assist researchers with the evaluation of research co-design. Fifty-one included reviews reported on fifteen evaluation themes, which were grouped into the following clusters: People (within co-design group), group processes, research processes, co-design context, people (outside co-design group), system and sustainment. Most reviews did not report measurement methods for the evaluation themes. If methods were mentioned, they mostly included qualitative data, informal consumer feedback and researchers’ reflections. This finding strengthens our argument that a framework may be helpful in supporting methodologically robust studies to assess co-design processes and impacts. The Co-Design Evaluation Framework has adopted a tree metaphor. It presents the processes and people in the co-design group (below-ground) as the underpinning system- and people-level outcomes beyond the co-design group (above-ground). To evaluate stakeholder involvement in research, researchers may wish to consider any or all components in the tree. Which, and how many, components are appropriate to evaluate depends on the nature of the co-design approach and the key questions that stakeholders aim to address. Nonetheless, it will be important that evaluations delineate what parts of the research project have incorporated a co-design approach.

The Equator reporting checklist for Research Co-Design, GRIPP2, provides researchers with a series of concepts that should be considered and reported on when incorporating patient and public involvement in research [10]. These concepts include, but are not limited to, methods of involving patients and the public in research and intensity of engagement. The Co-Design Evaluation Framework is not intended as a replacement for the GRIPP2, rather, it can be used prospectively to inform development of the co-design project or retropsectively to inform completion of the GRIPP2. Table 7 provides hypothetical examples of research questions that co-design evaluation projects might address. The framework could be used at multiple points within co-design projects, including prospectively (planning for evaluation before the co-design process has started), concurrently ( incorporating improvements during the co-design process) and retrospectively (reviewing past co-design efforts to inform future projects).

**Table 7:** Example questions to consider for prospective, concurrent and retrospective use of the framework

	**General example questions to consider**	**Specific evaluation theme example questions to consider**
**Prospective**	• Which evaluation themes are important to our group? • How will those themes be evaluated? • When will the evaluation take place? • Who are the participants in the evaluation?	• *Structure and composition of the co-design group*: Who should be part of the co-design group so that relevant lived experiences and areas of expertise are included? • *Decision making process:* How can co-design members be supported to equally engage in the decision making process? And how can we evaluate that?
**Concurrent**	• Now that we have started our evaluation, what are the most important signals that we should respond to now, by changing our co-design process? • Now that we have identified some room for improvement in our co-design process, what expertise can we draw on to decide how to improve? (this might include consumer expertise) • Do other/additional evaluation themes need to be included in subsequent evaluation moments?	• *Level/quality of engagement:* What activities are the co-design members involved in and what is the attendance rate for the co-design sessions/meetings? • *Emotional factors:* How do co-design members feel about being engaged in the co-design process?
**Retrospective**	• Now that we are at the end of the study, is there evidence that the co-design process and/or product (e.g. if an intervention was designed) could be improved next time? • What expertise can we draw on to decide how to improve future co-design activities? • Do we have evidence that other/additional evaluation themes need to be included in future co-design projects?	• *Healthcare system level outcomes*: Is there evidence that the co-designed outcome/ intervention/ innovation has been adopted within the targeted healthcare context? If yes, what was its reach? • *Patient and community outcomes*: Did the co-design approach influence people in the community? If yes, what patient and community outcomes were achieved?

Our systematic overview of reviews identified multiple evaluation themes. Some of these overlapped with reported values associated with public involvement in research [80], community engagement measures [15] and reported impacts of patient and public involvement in research, as described by others [16, 81, 82]. The added value of our systematic overview of reviews is that we went beyond a list of items and took it one step further by looking at evaluation themes, potential associations between evaluation themes, clusters of evaluation themes and ultimately developed a framework to assist others with research co-design evaluation.

Some reviews in our overview of reviews proposed potential associations between evaluation themes. Yet, these proposed associations were not empirically tested. One of the included studies [58] proposed conditions and mechanisms involved in co-design processes and outcomes related to diabetes research. Although it is a promising starting point, this should be further explored. A realist evaluation including other research topics and other approaches, such as the use of logic models, which was also recognised in the updated MRC framework [9], might help to build on explorations of included mechanisms of action [83] and give insight into how core ingredients contribute to certain co-design processes and outcomes. As recognised by others [6, 84], the reporting practice of research co-design in the literature could be improved as details about context, mechanisms and expected outcomes are frequently missing. This will also help us to gain a better understanding of what works for whom, why, how and in which circumstances.

The lack of a consistent definition of co-design makes it challenging to identify and synthesise the literature, as recognised by others [6]. Given that there are so many different terms used in the literature, there is a risk that we might have missed some relevant papers in our overview of reviews. Nevertheless, we tried to capture as many as possible synonyms of co-design in our search terms. The absence of quality assessment of included studies in our overview of reviews can be seen as a limitation. However, our overview of reviews did not aim to assess existing literature on the co-design process, but rather focused on what to evaluate, how and when. We did note whether the reported evaluation themes were based on empirical evidence or authors’ opinions. Primary studies reported in the included reviews were not individually reviewed as this was outside the scope of this paper. A strength in our methods was the cyclical process undertaken between steps 1 and 2. Analysis of the data extracted from the overview was refined over three phases following rigorous discussions with a diverse and experienced stakeholder panel. It was a strength of our project that a mix of stakeholders were involved, including consumers, healthcare professionals and researchers.

Stakeholders are frequently engaged in research but if research co-design processes and outcomes are not evaluated, there will be limited learning from past experiences. Evaluation is essential to make refinements during existing projects and improve future co-design activities. It is also critical for ensuring commitments to the underpinning values of c-odesign are embedded within activities.

A systematic review of all primary studies within the included reviews of this overview of reviews, would allow greater depth relating to the practicalities of how to evaluate certain themes. It would lead to a better understanding of existing measures and methods and which evaluation areas need further development. Future research should also focus on whether co-design leads to better outcomes than no co-design (only researcher-driven research). To our knowledge, this has not been explored yet. Moreover, future research could gain better insight into the mechanisms of change within co-design and explore potential associations between evaluation themes for example, those proposed in the included reviews between level/quality of engagement and emotional factors.

## Conclusion

We followed a systematic, iterative approach to develop a Co-Design Evaluation Framework that can be applied to various phases of the research co-design process. Testing of the utility of the framework is an important next step. We propose that the framework could be used at multiple points within co-design projects, including prospectively (planning for evaluation before the co-design process has started), concurrently (to incorporate improvements during the co-design process) and retrospectively (reviewing past co-design efforts to inform future projects).

## Supplementary Information

Supplementary Material 1.

Supplementary Material 2.

Supplementary Material 3.

Supplementary Material 4.

## Data Availability

All data generated during this study are included either within the text or as a supplementary file.

## Abbreviations

MRC: Medical Research Council
GRIPP: Guidance for Reporting Involvement of Patients and the Public
