# Supplementary material for: Evaluation of research co-design in health: a systematic overview of reviews and development of a framework
Source: Implement Sci. 2024 Sep 11;19:63. doi: 10.1186/s13012-024-01394-4 (PMC11391618; doi:10.1186/s13012-024-01394-4)
Supplement: Supplementary file 2 — Supplementary Material 2. [file 13012_2024_1394_MOESM2_ESM.docx]

**Additional file 2:** MEDLINE (Ovid) search string (based on Slattery et al., 2020; Greenhalgh et al.,2019; Vat et al., 2019)

| Search # | String |
| --- | --- |
| 1 | ((Patient* or User* or Clinician* or Provider* or Caregiver* or carer or family or Public or Communit* or Stakeholder* or Consumer* or client* or health* professional* or health* worker* or allied health* or lay person or citizen or Payer or Purchaser or Insurer or Employer or Pharmaceutical or Hospital or health system or health organi?ation or funder) adj3 (Collaborat* or Consult* or input or Engag* or Involv* or Design* or Participat* or Partner* or Develop* or produc* or co-produc* or co produc* or co-design or co design or codesign or dialog or opinion* or empower or inform*)).ti. and (research* or study or trial* or pilot).tw and (health*).tw |
| 2 | (exp Patient Participation/ or exp Community Participation/ or exp community-based participatory research/) and (research* or study or trial* or pilot).ti. |
| 3 | 1 or 2 or (research adj3 (co-design or co design or codesign) adj3 health*).ti |
| 4 | 3 and (outcome* or evaluat* or impact* or effect* or measurement* or metrics or assess* or framework* or criteria or indicator*).tw. |
| 5 | META-ANALYSIS/ |
| 6 | META-ANALYSIS AS TOPIC/ |
| 7 | (meta analy* or metaanaly*).tw. or (systematic* adj2 (review* or overview* or search*)).tw. or (literature adj2 (review or search*)).tw. or (medline or pubmed or cochrane or embase or cinahl or cinahl or lilacs or science citation index or "web of science" or conference proceedings or psyclit or psychlit or psycinfo or psychinfo).ab. or (additional adj (papers or articles or sources)).ab. or (search term* or published articles or search strateg*).ab. or reference list*.ab. or (electronic adj (sources or resources or databases)).ab. or (bibliograph* or handsearch* or hand search* or manual* search*).ab. or (relevant adj (journals or articles)).ab. |
| 8 | REVIEW LITERATURE AS TOPIC/ |
| 9 | 5 or 6 or 7 or 8 |
| 10 | (Review or systematic reviews).pt. |
| 11 | exp CLINICAL TRIALS AS TOPIC/ |
| 12 | RANDOMIZED CONTROLLED TRIALS/ |
| 13 | (data adj2 (extract* or analys*)).ab. or (selection criteria or critical appraisal).ab. or ((randomi* or controlled or cohort* or observational or retrospective* or nonrandomi* or case*) adj2 (trial* or stud*)).ab. |
| 14 | 11 or 12 or 13 |
| 15 | 10 and 14 |
| 16 | 9 or 15 |
| 17 | COMMENT/ or LETTER/ or EDITORIAL/ |
| 18 | 16 not 17 |
| 19 | ANIMALS/ not (ANIMALS/ and HUMANS/) |
| 20 | 18 not 19 |
| 21 | 4 and 20 |
| 22 | limit 21 to yr="2000 -Current" |

Lines 5 to 20 of Table 1 are an adaptation of the SIGN systematic review search filter (https://www.sign.ac.uk/what-we-do/methodology/search-filters/)
