# Supplementary material for: Evaluation of research co-design in health: a systematic overview of reviews and development of a framework
Source: Implement Sci. 2024 Sep 11;19:63. doi: 10.1186/s13012-024-01394-4 (PMC11391618; doi:10.1186/s13012-024-01394-4)
Supplement: Supplementary file 3 — Supplementary Material 3. [file 13012_2024_1394_MOESM3_ESM.docx]

**Additional file 3:** Reported evaluation themes, evaluation methods and proposed associations between evaluation themes

| **Reference** | **Participants in the evaluation** | **Timing of evaluation** (e.g. pre, mid and/or post co-design) | **Reported evaluation themes** | **Study design and data collection methods** (formally assessed, participant experiences (e.g. informal feedback), authors’ hypotheses or not reported) | **Reported proposed associations between evaluation themes and example quotes**  (formally assessed, participant experiences (e.g. informal feedback), authors’ hypotheses or not reported) |
| --- | --- | --- | --- | --- | --- |
| **Baldwin et al. (2018)** | Older people co-researchers and academic researchers | Not reported | - Structure and composition of the co-design group - Contextual enablers/barriers - Interrelationships between group members - Emotional factors - Cognitive factors - Level/quality of engagement - Research process - Patient and community outcomes | Interviews (n=6), focus groups (n=2), reflection meetings (n=3), co-researcher narrative reflections (n=1), field notes (n=2) and document reviews (n=2). In 3 studies, an external evaluator collected the data. | Not reported |
| **Bench et al. (2018)** | Staff, patient and carer participants and facilitators | Not reported | - Contextual enablers/barriers   • Interrelationships between group members • Emotional factors • Level/quality of engagement • Research process  • Patient and community outcomes | Ethnography (n=1), including observations, interviews and focus groups, and evaluation forms with open-ended questions (n=1) | Not reported |
| **Bethell et al. (2018)** | Not reported | Before and after in some cases but in other cases not reported. | - Structure and composition of the co-design group - Contextual enablers/barriers - Interrelationships between group members - Emotional factors - Cognitive factors - Level/quality of engagement - Research process | Two before and after studies and one qualitative study (no details reported) | Not reported |
| **Brett et al. (2014)** | Not reported | Not reported | - Structure and composition of the co-design group - Contextual enablers/barriers   • Interrelationships between group members • Emotional factors • Cognitive factors   - Value-proposition   • Level/quality of engagement • Research process  • Patient and community outcomes | 42 were qualitative studies, 12 were case studies, three were a case series and three were cross-sectional studies | *“The lack of preparation and training of the service users* **(=cognitive factors)** *left them feeling inexperienced* **(=emotional factors)** *and unable to contribute in group situations, which in turn led to low attendance rates throughout one study”* (p. 390) **(=level/quality of engagement).**  *“Researchers could be sceptical about PPI* **(=emotional factors)***, leading to a lack of commitment and a tokenistic attitude towards involving users in their research”* (p. 390) **(=level/quality of engagement)**. |
| **Di Lorito et al. (2017)** | Not reported | Not reported | • Structure and composition of the co-design group  • Interrelationships between group members • Emotional factors • Cognitive factors • Research process  • Patient and community outcomes | Not reported  (the reported outcomes are proposed as potential outcomes) | Not reported |
| **Domecq et al. (2014)** | Not reported | Not reported | • Emotional factors • Research process | Not reported | Not reported |
| **Fox et al. (2021)** | Not reported | Not reported | • Structure and composition of the co-design group • Interrelationships between group members • Emotional factors • Cognitive factors • Research process  • Patient and community outcomes | Participant experiences (e.g. informal feedback) +  authors’ hypotheses | Not reported |
| **Frankena et al. (2015)** | People with intellectual disabilities and researchers | Not reported | • Interrelationships between group members • Emotional factors • Cognitive factors • Research process  • Healthcare professional-level outcomes | Participant experiences (e.g. informal feedback) +  authors’ hypotheses | Not reported |
| **Fudge et al. (2007)** | Older people, professionals and researchers | Not reported | • Interrelationships between group members • Emotional factors • Cognitive factors • Research process • Patient and community outcomes | Formally assessed + participant experiences (e.g. informal feedback) | Not reported |
| **George et al. (2015)** | Not reported | Not reported | • Patient and community outcomes | Not reported | Not reported |
| **Legare et al. (2011)** | Not reported | Not reported | • Interrelationships between group members • Emotional factors • Cognitive factors | Not reported | Not reported |
| **Manafo et al. (2018)** | Patients and researchers | Not reported | • Interrelationships between group members • Emotional factors • Cognitive factors • Research process | Not reported | Not reported |
| **McCarron et al. (2021)** | Patients and researchers | Not reported | • Interrelationships between group members   - Decision making process - Cognitive factors - Research process - Patient and community outcomes | Not reported | Not reported |
| **Miller et al. (2012)** | Community members | Not reported | • Emotional factors • Cognitive factors • Sustainment of the co-design team or activities • Patient and community outcomes | Not reported | Not reported |
| **Shen et al. (2017)** | Parents and researchers | Post | • Structure and composition of the co-design group   - Decision making process - Research process - Patient and community outcomes | Not reported | Not reported |
| **Vat et al. (2020)** | Patients and researchers | Not reported | • Emotional factors • Research process  • Healthcare system level outcomes   - Organisational level outcomes - Patient and community outcomes | Not reported | Not reported |
| **Velvin et al. (2022)** | Patients and researchers | Not reported | • Emotional factors • Cognitive factors • Level/quality of engagement • Research process | Not reported | Not reported |
| **Wiles et al. (2022)** | Consumers and researchers | Not reported | • Emotional factors • Cognitive factors • Level/quality of engagement • Research process | Not reported | Not reported |
| **Cook et al. (2019)** | Not reported | Not reported | - Contextual enablers/barriers - Emotional factors - Research process - Sustainment of the co-design team or activities - Patient and community outcomes | Authors’ hypotheses | Not reported |
| **Chambers et al. (2019)** | Patients, clinicians, carers and academics | Not reported | • Interrelationships between group members • Emotional factors • Cognitive factors • Level/quality of engagement • Research process  • Sustainment of the co-design team or activities | Not reported | Not reported |
| **Brett et al. (2012)** | Not reported | Not reported | • Level/quality of engagement  • Decision making process   - Emotional factors   • Research process | Not reported | Not reported |
| **Boaz et al. (2015)** | Not reported | Not reported | • Cognitive factors • Research process  • Healthcare professional-level outcomes   - Healthcare system level outcomes - Organisational level outcomes - Patient and community outcomes | Not reported | Not reported |
| **Boivin et al. (2018)** | Not reported | Not reported | • Level/quality of engagement • Research process  • Patient and community outcomes | Not reported | Not reported |
| **Anderst et al. (2020)** | Not reported | Not reported | • Structure and composition of the co-design group   - Contextual enablers/barriers   • Interrelationships between group members   - Decision making process - Emotional factors - Cognitive factors - Research process - Healthcare professional-level outcomes   • Patient and community outcomes | Not reported | Not reported |
| **Arnstein et al. (2020)** | Not reported | Not reported | • Structure and composition of the co-design group   - Contextual enablers/barriers   • Interrelationships between group members   - Decision making process   • Emotional factors • Cognitive factors  • Research process | Not reported | Not reported |
| **Becerril-Montekio et al. (2022)** | Not reported | Not reported | • Structure and composition of the co-design group  • Level/quality of engagement • Healthcare professional-level outcomes   - Healthcare system level outcomes - Organisational level outcomes | Not reported | *“The identification of the different terms and approaches to the close collaboration of health staff and  decision-makers with professional researchers is essential to promote its effective application and influence on  the utilization of evidence. Yet, it is also necessary to insist in their co-participation throughout the whole investigation process* **(=level/quality of engagement)** as a relevant way to improve research results uptake, strengthen health systems and advance towards universal health coverage” (p. 2) **(=healthcare system level outcomes)**. |
| **Bird et al. (2020)** | Not reported | Not reported | • Structure and composition of the co-design group   - Contextual enablers/barriers   • Interrelationships between group members   - Decision making process   • Cognitive factors • Research process | Not reported | Not reported |
| **Dawson et al. (2018)** | Not reported | Not reported | • Structure and composition of the co-design group  • Cognitive factors • Research process | Not reported | Not reported |
| **Harris et al. (2019)** | Not reported | Not reported | - Structure and composition of the co-design group - Contextual enablers/barriers - Interrelationships between group members - Decision making process - Emotional factors - Cognitive factors - Value-proposition - Level/quality of engagement - Research process - Sustainment of the co-design team or activities - Patient and community outcomes | Not reported | *“Involvement may be influenced by a number of aspects of the surrounding context. For example, researchers may be reluctant to involve people* **(=level/quality of engagement)** *because they cannot see the value of it, they may feel that the time needed cannot justify the expense* **(=value-proposition)** *and they may feel unequipped with the requisite skills for engagement.”* (p. 909) **(=cognitive factors)**  *“People with diabetes may lack confidence to participate* **(=emotional factors)** *because they do not understand research or recognize the value of their lived experience* **(=cognitive factors).** These attitudes and feelings about engagement act as mechanisms, which either enable or constrain involvement in research.” (p. 909) **(=level/quality of research)**  *“The different approaches to targeted involvement suggested that frequent involvement* **(=level/quality of engagement)** *establishes/strengthens relationships* **(=interrelationships between group members)** *and iterative discussions can produce consensus on research priorities as well as useful patient information.”* (p. 914) **(=decision making process)**  *“In contexts where researchers are able to relinquish control, reciprocal relationships can emerge* **(=decision making process),** *triggering mechanisms where users feel confident to contribute knowledge* **(=emotional factors),** *thereby increasing the relevance of the research.”* (p. 914) **(=research process)**  *“This review found that projects which promote frequent and regular contact across researchers, patients with diabetes and wider communities* **(=level/quality of engagement)** *are able to develop reciprocal relationships* **(=interrelationships between group members)** *where the lived experiences of people are instrumental in developing and conducting relevant and accessible interventions promoting diabetes self‐management.”* (p. 915) **(=research process)**  *“In contexts where researchers are able to share control and ownership* **(=decision making process),** *community advisory groups and community researchers can achieve and sustain high rates of participation.”* (p. 915) **(=level/quality of engagement)**  *“When setting priorities, the involvement of people with experience of diabetes may increase relevance* **(=research process)** *but only if there is a context where: there is productive and sufficiently long interaction between researchers and patients/communities* **(=level/quality of engagement)**; *there is facilitation and/or training providing opportunities for people to share knowledge and experiences* **(=cognitive factors)**; *the people involved are similar or the same as those that will be designing the intervention* **(=structure and composition of the co-design group).** *When these conditions exist, mechanisms are triggered where: researchers aware of their own stance and are willing to relinquish control; community members feel safe to share concerns and disagree; people commit to working out differences* **(=decision making process).** *The outcomes of the priority setting process are as follows:* problem framing which allows patient and community concerns to be foregrounded; agreed topics that are taken to funding bodies **(=research process)**; raised awareness of health issues and possible solutions that are relevant to patients/communities **(=patient and community outcomes)**; continued interest in participating in design and mobilizing to take action.” (p. 914) **(=level/quality of engagement)**  *“During the design stage, the same context is important, with the added provisos that: relevant stakeholders and agencies need to be included* **(=structure and composition of the co-design group);** *stakeholders need experience in facilitating partnership working* **(=interrelationships between group members);** *safe and comfortable spaces need to be identified to encourage participation of new stakeholders* **(=contextual enablers/barriers).** *If these conditions are in place, then members of the project group will feel:* *empowered to judge the feasibility and appropriateness of the emerging design* **(=emotional factors);** *clear on who has the expertise to undertake different study tasks* **(=cognitive factors).** *If successful, the end products are more culturally acceptable interventions, more appropriate approaches to recruitment and more user-friendly information and tools. Excluding people from the design process may lead to project information that is difficult to understand and less culturally acceptable.”* (p. 914) **(=research process)** |
| **Hubbard et al. (2008)** | Not reported | Not reported | - Structure and composition of the co-design group - Contextual enablers/barriers   • Interrelationships between group members   - Decision making process - Emotional factors - Cognitive factors - Level/quality of engagement - Research process - Patient and community outcomes | Not reported | Not reported |
| **Hubbard et al. (2007)** | People affected by cancer | Not reported | • Structure and composition of the co-design group   - Contextual enablers/barriers   • Decision making process  • Emotional factors  • Cognitive factors • Research process | Not reported | Not reported |
| **Jones et al. (2015)** | Surgical patients | Not reported | • Research process | Not reported | Not reported |
| **Drahota et al. (2016)** | Not reported | Not reported | • Interrelationships between group members  • Cognitive factors • Research process  • Sustainment of the co-design team or activities • Patient and community outcomes   - Organisational level outcomes | Not reported | Not reported |
| **Forsythe et al. (2014)** | Not reported | Not reported | • Structure and composition of the co-design group  • Interrelationships between group members   - Cognitive factors - Research process - Sustainment of the co-design team or activities - Patient and community outcomes | Not reported | Not reported |
| **Di Lorito et al. (2018)** | Co-researchers and academics | Not reported | • Structure and composition of the co-design group  • Interrelationships between group members  • Emotional factors  • Cognitive factors  • Research process | Not reported | Not reported |
| **Lander et al. (2014)** | Not reported | Not reported | • Emotional factors  • Patient and community outcomes | Not reported | Not reported |
| **Lee et al. (2017)** | Not reported | Not reported | • Research process   - Healthcare professional-level outcomes | Not reported | Not reported |
| **Malterud et al. (2020)** | Not reported | Not reported | - Contextual enablers/barriers   • Interrelationships between group members   - Decision making process   • Emotional factors  • Cognitive factors  • Patient and community outcomes | Formally assessed + participant experiences (e.g. informal feedback) | Not reported |
| **Miah et al. (2019)** | People with dementia, academics | Not reported | • Cognitive factors  • Research process | Participant experiences (e.g. informal feedback) | Not reported |
| **Nilsen et al. (2006)** | Not reported | Not reported | - Research process - Healthcare professional-level outcomes - Patient and community outcomes - Healthcare system level outcomes - Organisational level outcomes | Not reported | Not reported |
| **Oliver et al. (2004)** | Not reported | Not reported | • Structure and composition of the co-design group   - Interrelationships between group members - Decision making process - Contextual enablers/barriers   • Emotional factors   - Level/quality of engagement   • Research process   - Sustainment of the co-design team or activities | Not reported | *“Least was learnt from simple, written*  *consultations either in terms of consumers’ ideas and priorities for research or about the methods used to involve them possibly owing to the low response rates* **(=level/quality of engagement)**, *short timetables and lack of guidance or support for*  *consumers.”* (p. 33) **(=contextual enablers/barriers)**  *“Empowerment* **(=emotional factors)** *and partnership* **(=interrelationships between group members)** *benefited consumer involvement.”* (p. 39) **(=level/quality of engagement)** |
| **Orlowski et al. (2015)** | Not reported | Not reported | • Structure and composition of the co-design group   - Interrelationships between group members - Decision making process   • Cognitive factors  • Research process | Not reported | Not reported |
| **Pii et al. (2019)** | Not reported | Not reported | - Decision making process   • Emotional factors  • Cognitive factors  • Research process | Not reported | Not reported |
| **Sandoval et al. (2012)** | Not reported | Not reported | • Interrelationships between group members   - Decision making process   • Emotional factors  • Patient and community outcomes   - Healthcare system level outcomes - Organisational level outcomes | Not reported | Not reported |
| **Sangill et al. (2019)** | Not reported | Not reported | • Interrelationships between group members   - Decision making process - Contextual enablers/barriers   • Emotional factors  • Cognitive factors   - Level/quality of engagement - Research process | Not reported | *“Some studies reported that higher levels of user involvement* **(=level/quality of engagement)** *were more likely to achieve recruitment success.”* (p. 808) **(=research process)**  *“Tokenism* **(=level/quality of engagement)** *was reported to be linked to user-researchers’ lack of research training* **(=cognitive factors)** *and uncertainty about how they were supposed to contribute* **(=emotional factors).** *This positioned the academic researcher as the research authority, as someone who always knows*  *what was right, and positioned the user-researchers as someone not in complete control, in need of a legitimate voice, expertise, and self-confidence.”* (p. 810) **(=interrelationships between group members)**  *“When user-researchers did not get feedback or experienced a lack*  *of interest from academic researchers* **(=interrelationships between group members),** *it could obstruct more extensive involvement in the research processes.”* (p. 810) **(=level/quality of engagement)** |
| **Schelven et al. (2020)** | Not reported | Not reported | • Structure and composition of the co-design group   - Interrelationships between group members - Decision making process - Emotional factors - Cognitive factors - Research process | Not reported | Not reported |
| **Schilling et al. (2017)** | Not reported | Not reported | • Structure and composition of the co-design group   - Contextual enablers/barriers - Interrelationships between group members - Decision making process - Emotional factors - Cognitive factors - Research process | Not reported | Not reported |
| **Shippee et al. (2013)** | Not reported | Not reported | • Structure and composition of the co-design group  • Interrelationships between group members   - Decision making process   • Research process  • Cognitive factors | Not reported | *“Co-learning* **(=cognitive factors)** *also may increase patients and service users’ confidence* **(=emotional factors),** *promoting more active engagement and reducing the risk of tokenistic patient and service users’ involvement.”* (p. 1156) **(=level/quality of engagement)** |
| **Vaughn et al. (2017)** | Immigrants | Not reported | • Interrelationships between group members   - Decision making process   • Emotional factors  • Cognitive factors   - Research process - Health outcomes for co-design group   • Patient and community outcomes   - Healthcare system level outcomes - Organisational level outcomes | Not reported | Not reported |
| **Walmsley et al. (2018)** | People with an intellectual disability | Not reported | • Interrelationships between group members  • Emotional factors  • Cognitive factors  • Research process  • Patient and community outcomes | Not reported | Not reported |
| **Weschke et al. (2022)** | Patients | Not reported | • Structure and composition of the co-design group   - Contextual enablers/barriers   • Research process | Not reported | Not reported |
