# Supplementary figures and images for: Evaluation of research co-design in health: a systematic overview of reviews and development of a framework
Source: Implement Sci. 2024 Sep 11;19:63. doi: 10.1186/s13012-024-01394-4 (PMC11391618; doi:10.1186/s13012-024-01394-4)

**Additional file 4: Two draft options of the research co-design evaluation framework**

| **Draft 1** | **Draft 2** |
| --- | --- |
| 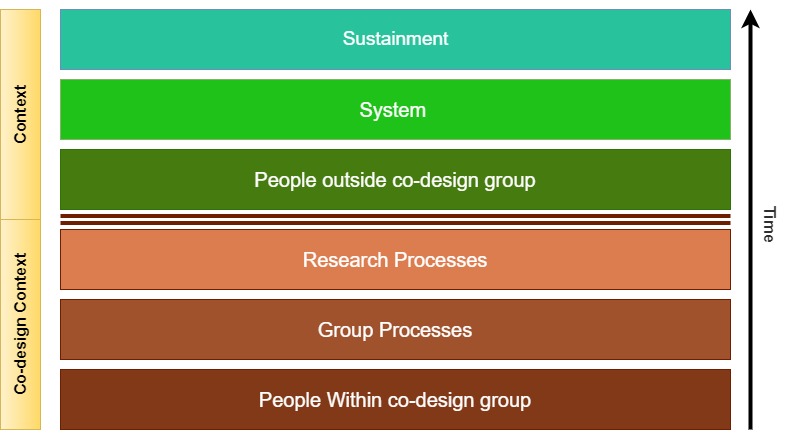 | 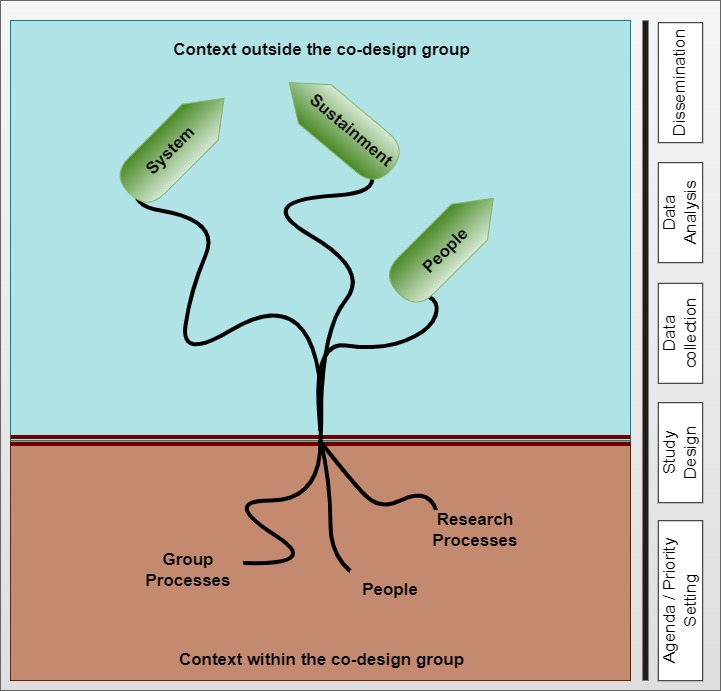 |

Supplement: Supplementary file 4 — Supplementary Material 4. [file 13012_2024_1394_MOESM4_ESM.docx]
